# Severe infections in giant cell arteritis - incidence over time and relation to large vessel involvement and comorbidities, a population-based study

**DOI:** 10.1186/s41927-026-00676-2

**Published:** 2026-07-22

**Authors:** Nazanin Naderi, Karin Wadström, Ulf Bergström, Aladdin J. Mohammad, Carl Turesson

**Affiliations:** 1 Section of Rheumatology, Örnsköldsvik Hospital, Örnsköldsvik, Sweden; 2 Rheumatology, Department of Clinical Sciences, Malmö, Lund University, Jan Waldenströms gata 1B, Malmö, 205 02 Sweden; 3 Center for Rheumatology, Academic Specialist Center, Region Stockholm, Stockholm, Sweden; 4 Department of Rheumatology, Skåne University Hospital, Malmö and Lund, Sweden; 5 Rheumatology, Department of Clinical Sciences, Lund, Lund University, Lund, Sweden; 6 Department of Medicine, University of Cambridge, Cambridge, UK

**Keywords:** Giant cell arteritis, Severe infections, Risk factors, Prognostic factors, Predictors, Large vessel involvement

## Abstract

**Background:**

Patients with giant cell arteritis (GCA) are considered to be at increased risk of infections. The objectives of this study were to investigate the risk of severe infections in different time intervals after the diagnosis of GCA, compared to the general population, and to explore potential predictors for severe infections including large vessel involvement (LVI).

**Methods:**

Patients with biopsy-proven GCA, diagnosed between 2002 and 2010 in Region Skåne, Sweden, were identified and each case compared with 4 age-, sex- and residence area-matched reference subjects. Aortic involvement and other LVI was identified by case record review. Data on severe infections (requiring hospitalization) were obtained from linkage to the Skåne Healthcare Register through 2011. Prevalent comorbidities at GCA diagnosis were identified using ICD-10 codes. Predictors of severe infection in patients with GCA were evaluated using Cox regression.

**Results:**

In 516 patients with GCA (100 with LVI), 22.9% experienced severe infections (mainly pneumonia and urinary tract infections), incidence rate of severe infection episodes 9.65/100 person-years (py). The incidence was particularly high during the first six months after diagnosis (14.8/100 py, rate ratio compared to reference subjects 3.27; 95% CI 2.06; 5.11). Higher age, LVI and several pre-existing comorbidities were associated with severe infections. In multivariable analysis, aortic involvement was a significant predictor of severe infection (multi-adjusted hazard ratio 1.75; 95% CI 1.04; 2.94).

**Conclusions:**

Compared to reference subjects from background population, patients with biopsy-positive GCA have increased risk of severe infections, in particular in early disease. Age, comorbidities and aortic involvement may predict severe infections in GCA. Further prospective studies on this subject, including detailed assessment of LVI, are needed.

**Supplementary Information:**

The online version contains supplementary material available at 10.1186/s41927-026-00676-2.

## Background

Giant cell arteritis (GCA), a vasculitis of large and medium-sized vessels, is the most common type of vasculitis in adults aged 50 years or older [1] with a female predominance of 2–3:1 [1]. GCA can afflict the aorta and its main distributary branches [2, 3].

The disease is associated with morbidities related both to the vasculitis itself as well as its treatment with glucocorticoids (GC).

Some comorbidities have been reported to be significantly more common in patients with GCA compared to the general population, e.g. cardiovascular disease (CVD) manifestations, i.e. strokes, thoracic aortic aneurysms and dissections, myocardial infarctions, and peripheral vascular disease [4, 5]. CVD related mortality has also been shown to be higher in GCA patients compared to the general population [6, 7].

Osteoporosis, hyperlipidemia, diabetes, cataracts, muscle atrophy, thrombosis and infections also appear in higher proportions in this patient group after diagnosis [8, 9], although the prevalence of diabetes has been shown to be lower before diagnosis [10, 11], suggesting that this comorbidity in patients with GCA is mainly driven by inflammation and/or GC treatment.

GC treatment has long been recognized as a risk factor for infections with varying severity, and the risk of infection increases with the dose and treatment duration of GC in patients with rheumatic autoimmune diseases. Although the excess risk is modest for those taking < 5 mg of prednisone equivalents per day, it has been reported to increase considerably with higher doses, reaching a relative risk of > 5 in those taking > 20 mg/day compared to patients with rheumatic autoimmune diseases not taking GC [12–16].

Most of these data are from studies of patients with chronic arthritis. Such patient populations are treated with GC doses at diagnosis well below those used in GCA.

To date, the main therapy for GCA consists of GC [9]. GC are used as induction therapy with common doses of 40–60 mg prednisolone/prednisolone equivalent, as well as maintenance therapy with targeted doses as low as possible, in best case scenarios cessation within two years. Patients who are not able to taper GC to acceptable doses, i.e. those with frequent relapses and/or with aggressive disease, receive add-on therapy with other immunosuppressants [17, 18].

Only a few studies have investigated the types of infections, possible time-dependent trends and association with background comorbidities during the course of GCA compared to the general population [19–22]. All but one [21] found increased risk of severe infections in the GCA population compared to the general population.

The presence of large vessel involvement (LVI) in GCA has in some studies been shown to be associated with higher relapse frequency [23–25]. Relapses require escalation of the GC dose with prolonged course of GC treatment leading to an increased cumulative GC dose. Furthermore, the cumulative disease activity in patients with GCA and LVI may per se affect the risk of severe infections. To the best of our knowledge, there have been no studies investigating the relation between LVI in GCA and the risk of severe infections.

The aims of this study were to: (I) investigate the pattern of severe infections in patients with GCA, (II) compare the risk of infection and the burden of infections in different time intervals after GCA diagnosis to the general population, (III) assess the impact of age, sex, LVI and prevalent comorbidities on infection risk in patients with GCA.

## Methods

### Patients and reference subjects

Patients with biopsy-proven GCA, diagnosed between January 1, 2002 and December 31, 2010, in Skåne, the southernmost region in Sweden, were identified through the common database for pathology departments in Skåne, and a structured review of the pathology reports, as previously described [26]. For each patient, 4 reference subjects from the general population were identified matched for sex as well as age and area of residency at the index date (corresponding to the date of diagnosis of the matched case). Reference subjects were retrieved from the Skåne Healthcare Register (SHR). Information on time of death or migration from the region, based on register linkage enabled by personal identification numbers, was retrieved from the Total Population Register via Region Skåne. The time of follow-up was from the date of diagnosis of GCA (index date for reference subjects), to death, migration from the region or end of the study December 31, 2011, whichever came first.

### Potential predictors – LVI and glucocorticosteroids

The reports of all relevant radiological and clinico-physiological studies in patients with GCA were collected and reviewed according to a structured protocol by one of the authors (NN). LVI was defined and recorded as described in a previous study [27]. In short, LVI was defined as presence of aneurysm, ectasia, dissection or stenosis of the aorta and/or its main branches identified through any imaging method including ultrasound. In addition, positive 18-fluoro-2-deoxy-d-glucose positron emission tomography–computed tomography (18-FDG PET-CT) or other nuclear imaging methods indicating vasculitis, as assessed by the radiologist and/ or the clinical physiologist was also defined as LVI in the present study. LVI findings after GCA diagnosis or ≤ 1 year before the diagnosis were included in the study. Those with such signs of LVI were considered to have the LVI phenotype, regardless of the duration of GCA at the investigation. LVI overall, and aortic involvement specifically, were investigated as potential predictors of severe infections. As aortic involvement has been reported to be particularly overrepresented in patients with GCA compared to age-sex matched reference subjects [27], this was assessed as a separate entity in the prediction models. In exploratory analyses, we investigated the relations for affected arterial territories (in addition to aorta also cranial, visceral and upper/lower extremity arteries) and for each subtype of LVI (aneurysm, ectasia, stenosis, dissection and positive PET-CT/MRI indicating vascular inflammation) with risk of severe infections.

In a separate review of all available medical records, data on the first dose of GC were retrieved. As patients were followed by various care providers, availability of medical records beyond the time of diagnosis was limited, and we were unable to retrieve data on GC treatment over time.

### Potential predictors – comorbidities

Prevalent comorbidities were defined, using ICD-10 codes obtained from the SHR, as hospitalization with either of comorbidities of interest within four years before the date of diagnosis of GCA (Supplementary Table 1). The SHR has a good coverage, with diagnoses assigned at nearly 100% of inpatient episodes during the study period, and available data indicate a good validity [28]. The comorbidities (coronary artery disease, heart failure, cerebrovascular disease, peripheral arterial disease, diabetes type I and II, chronic obstructive pulmonary disease, chronic kidney disease with renal failure, renal failure non-specified, hip fracture, vertebral fracture, wrist fracture and proximal humeral fracture) were chosen based on their conceived potential of causing susceptibility to infections.

Potential associations for each comorbidity with severe infections were assessed separately. In addition, the Charlson comorbidity index (CCI) [29] was calculated for each patient, and patients were classified into the categories of CCI 0 (reference), 1, 2 and ≥ 3.

### Outcome – severe infections

Data regarding the presence and time of severe infections (requiring hospitalization) was obtained from linkage of the cohort of biopsy-proven GCA and the matched references to the SHR. Using ICD-10 codes, twenty categories of infections were defined (Supplementary Table 2).

### Statistics

Incidence rates (IRs) of severe infections in patients with GCA and reference subjects were estimated separately in each cohort using the events of severe infection as numerator and the total follow-up time as denominator. The follow-up time was calculated from date of GCA diagnosis (or index date for reference subjects) to death, migration from the region or end of study (December 31, 2011), whichever came first. IRs were stratified by follow-up time (0–6 months, 6–12 months, 1–2 years, 2–3 years, > 3 years), were estimated by dividing the number of severe infections in each time interval with the corresponding follow-up time. We also calculated IRs for the period preceding the diagnosis/index date (from the date of first available data in the SHR, i.e. January 1, 1998), and for the entire period after the diagnosis/index date. Incidence rate ratios (IRRs) of severe infections, stratified by category of follow-up time, were calculated for patients with GCA vs. reference subjects.

As previous severe infections in a patient may influence future hospitalizations, and to avoid multiple registration of the same inpatient event, due to one or more moves between different wards (a frequent occurrence during the study period), calculations of rates and ratios were done also including only the first severe infection per time interval (with censoring at first infection, death, migration from the region or end of study December 31 2011).

The 95% confidence intervals (CI) for IRs and IRRs were estimated using the Poisson distribution ratio.

Cox regression analysis was used to identify possible predictors of severe infections among patients with GCA. Patients were followed from time of GCA diagnosis to first severe infection or censoring at death, migration from the region or the end of the study period (December 31, 2011), whichever came first. The potential predictors of interest were age, sex, first prednisolone dose in mg at diagnosis, LVI status, prevalent comorbidities at diagnosis (categories as shown in Supplementary Table 1), category of CCI at diagnosis, as well as affected arterial territory and subtype of LVI (exploratory analyses). Crude and age-adjusted analyses were performed. Multivariable models included age and sex, as well as factors tending to show association (*p* < 0.10) in the unadjusted analyses. Spearman’s test was used to assess correlation between potential predictors. In cases with major co-linearity (*r* > 0.3 and *p* < 0.05), only the covariate with the strongest association in crude analysis was included in the multivariable model. As sensitivity analyses, we constructed alternative models including the variables that were excluded from the main model due to collinearity.

All statistical analyses were conducted with Statistical Package for the Social Sciences, SPSS V.25 for Windows (IBM SPSS Statistics).

## Results

### The GCA cohort and reference subjects, demographics

There were 516 patients (female 73%) with biopsy-proven GCA during the inclusion period (Table 1). The mean age at diagnosis was 75.3 years (yrs) [standard deviation (SD) 8.3]. These were compared to 2050 (female 73%) matched reference subjects. The mean follow-up time for GCA patients was 4.0 yrs [SD 2.7] and for the general population 4.7 yrs [SD 2.6].

**Table 1:** Characteristics and prevalent comorbidities at diagnosis in patients with biopsy-proven giant cell arteritis and reference subjects

		GCA cases	Reference subjects
*N*		516	2050
Age at diagnosis, mean [SD]		75.3 [8.30]	75.3 [8.28]
Female sex		377 (73.0)	1492 (72.8)
Prednisolone dose at diagnosis (mg), mean [SD]		49.9 [17.1]*	NA
Large vessel involvement		100 (19.4)	NA
Aortic involvement		62 (12)	NA
**Charlson Comorbidity index**	0	227 (43.8)	1558 (77.0)
	1	210 (40.7)	179 (8.7)
	2	43 (8.3)	162 (7.9)
	≥ 3	37 (7.2)	151 (7.4)
**Cardiovascular disease**	Coronary artery disease	62 (12.0)	375 (18.2)
	Heart failure	117 (22.7)	358 (17.5)
	Cerebrovascular disease	84 (16.3)	263 (12.8)
	Peripheral arterial disease	None	18 (0.9)
	CVD, any	189 (36.6)	663 (32.3)
**Diabetes mellitus**	Type I	26 (5.0)	109 (5.3)
	Type II	83 (16.1)	372 (18.1)
	DM, any	86 (16.7)	404 (19.7)
**Pulmonary disease**	Chronic obstructive pulmonary disease	54 (10.5)	170 (8.3)
**Kidney disease**	Chronic kidney disease with renal failure	17 (3.3)	56 (2.9)
	Renal failure, non-specified	17 (3.3)	52 (2.5)
**Malignancy**	Solid tumors	39 (7.6)	164 (8.0)
**Osteoporosis related fractures**	Hip fracture	54 (10.5)	172 (8.4)
	Vertebral fracture	12 (2.3)	33 (1.6)
	Wrist fracture	41 (7.9)	162 (7.9)
	Proximal humeral fracture	19 (3.7)	91 (4.4)

SD: standard deviation; CVD: cardiovascular disease; NA: Not applicable. Values are numbers and (percent) unless stated otherwise

* Missing data for 96 patients (19%)

### LVI status

At least one vascular imaging procedure was performed for 174 patients (33.7%). A total of 100 patients with GCA (19.4%; 25 men, 75 women) had LVI. These were on average younger compared to those without LVI (mean 73.2 yrs, SD 8.0, vs. 75.8 yrs, SD 8.3). Sixty-one of those with LVI (11.8% of all GCA patients; 14 men, 47 women) had aortic involvement (aneurysm, ectasia, dissection or vasculitis on 18-FDG PET-CT and or other nuclear imaging) with or without concomitant distributary vessel affection. Among patients with aortic involvement, 46 (8.9% of the GCA patients) had signs of involvement of one aortic segment, 8 (1.6%) of two aortic segments and 7 (1.4%) of all three aortic segments (ascending and descending thoracic aorta, and abdominal aorta). There were also patients with signs of vasculitis on 18-FDG PET-CT or other nuclear imaging with involvement of distributary arteries. Intracranial artery involvement was also noted (Supplementary Table 3).

### Comorbidities

The most prevalent comorbidity category (based on inpatient ICD codes within four years prior to GCA diagnosis) was CVD (36.6%) followed by diabetes (16.7%). The distribution of prevalent comorbidities at diagnosis in patients with GCA is shown in Table 1. The majority of the patients had a CCI of 0–1. Only 7% were in the highest CCI category (CCI ≥ 3). The higher CCI categories were driven by prior solid tumor malignancies.

### Incidence of severe infections

All patients with severe infections obtained from the linkage of the GCA cohort to the SHR were analyzed. A total of 221 severe infections were registered for 118 GCA patients (cumulative incidence 22.9%). There was a total of 6 deaths related to severe infections.

**Fig. 1:**
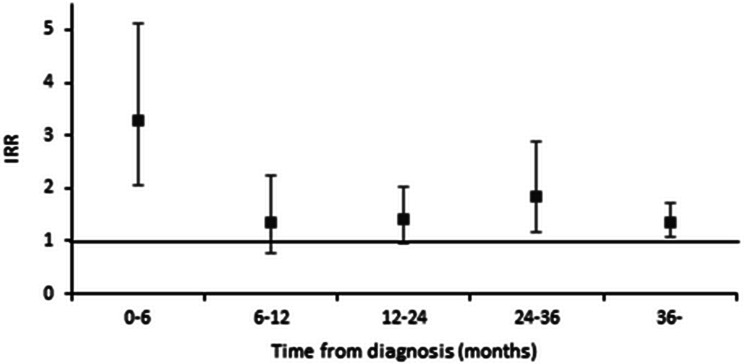
Incidence rate ratios (IRR), 95% with confidence intervals, for severe infections overall, in patients with GCA vs. reference subjects, by category of follow-up time

Forty-four patients (8.5%) experienced severe infections during the first year. The overall incidence rate was 9.65/100 person-years (py); (95% CI 8.42; 11.01). The incidence was highest during the first six months, 14.8/100 py (Table 2). During this period, the rate was significantly increased compared to reference subjects (IRR 3.27; 95% CI 2.06; 5.11), (Fig. 1).

**Table 2:** Rates of severe infections in patients with giant cell arteritis and reference subjects

		Cases			Reference subjects		
	Follow-up interval (months)	Infections (*n*)	Follow-up (py)	Rate/100 py (95% CI)	Infections (*n*)	Follow-up (py)	Rate/100 py (95% CI)
All severe infections	0–6	37	250.5	14.8 (10.4; 20.4)	45	995.9	4.5 (3.3; 6.1)
	6–12	21	240.2	8.7 (5.4; 13.4)	63	972.6	6.5 (5.0; 8.3)
	12–24	40	441.7	9.1 (6.5; 12.3)	116	1806.7	6.4 (5.3; 7.7)
	24–36	29	357.0	8.1 (5.4; 11.7)	65	1484.2	4.4 (3.4; 5.6)
	36-	95	1011.4	9.4 (7.6; 11.5)	287	4155.0	6.9 (6.1; 7.8)
First severe infection during the study period	Entire study period	118	2048.67	5.76 (4.77; 6.90)	279	9414.38	2.96 (2.63; 3.33)

n: number; py: person-years; CI: confidence interval

The rate of severe infections was numerically higher than that for the general population for each time interval during the entire follow-up, but the difference reached statistical significance only during the first six months and after > 2 years of follow-up, (Table 2; Fig. 1).

There was also a non-significant trend towards a higher incidence of severe infections before diagnosis in patients with GCA compared to reference subjects (IRR 1.23; 95% CI 0.98–1.54) and a significant difference for the entire follow-up after diagnosis (IRR 1.57; 95% CI 1.34–1.84) (Supplementary Table 6).

Patterns were similar for the incidence of first severe infection during each time interval (Supplementary Table 4, Supplementary Fig. 1). The incidence rate of first severe infection for the entire study period was increased in patients with GCA compared to the reference subjects (Table 2, IRR 1.94; 95% CI 1.55; 2.41).

### Distribution of pathogens and types of infection in patients with GCA

Inpatient records could be retrieved for 176/221 (79.6%) of the hospitalizations with ICD-10 codes for infection.

The majority of infections were caused by bacteria (Table 3). The single most common type of infection was bacterial pneumonia, accounting for 35.2% of all severe infections. There were 19 cases of septicemia. Viral infections were less frequent, the major infection type being gastroenteritis. Only one case of fungal infection was noted. There were no severe infections caused by mycobacteria or Pneumocystis jirovecii.

**Table 3:** Pathogens and type of infections in patients with giant cell arteritis

	*n* (%)
**Bacterial**	**163 (92.6)**
Pneumonia	62 (35.2)
Septicemia	19 (10.8) *
Meningitis	0
Bronchitis	2 (1.1)
Gastroenteritis	13 (7.4)
Cutaneous infection	13 (7.4)
Lower urinary tract infection	21 (11.9)
Upper urinary tract infection	2 (1.1)
Other	31 (17.6) #
**Viral**	**12 (6.8)**
Zoster	2 (1.1)
Gastroenteritis	5 (2.8)
Bronchitis	1 (0.6)
Other	4 (2.3) §
**Fungal**	**1 (1.1)**

* One patient with septicemia had also myelitis and endocarditis;

# Infection unspecified *n* = 24, Perianal abscess *n* = 2; Abdominal abscess *n* = 1, Sinusitis *n* = 1; Septic gonarthritis *n* = 1, Septic arthroplasty knee *n* = 1, Septic elbow bursitis *n* = 1

§ Acute upper respiratory tract infection *n* = 1, Influenza *n* = 1, Acute epiglottitis *n* = 1; Acute pharyngitis *n* = 1

The various types of pathogens verified by culture are presented in Supplementary Table 5.

### Predictors of severe infectious

Higher age at diagnosis of GCA was associated with future severe infections (Table 4). There was no significant association with oral GC dose at diagnosis, with or without age-adjustment.

**Table 4:** Cox regression analyses of potential predictors of severe infections in patients with GCA

		Crude HR (95% CI)		Age-adjusted HR (95% CI)
Age, per 5 years		1.42 (1.25–1.61)		NA
Male sex		1.38 (0.93–2.04)		1.47 (1.00–2.18)
Prednisolone dose at diagnosis (per SD)		1.11 (0.91–1.35)		1.13 (0.93–1.38)
Charlson comorbidity index	0	1 (ref)		1 (ref)
	1	0.89 (0.60–1.33)		0.83 (0.56–1.24)
	2	1.03 (0.51–2.09)		0.94 (0.47–1.92)
	≥ 3	1.74 (0.88–3.41)		1.48 (0.76–2.92)
Any large vessel involvement		1.30 (0.85–1.97)		**1.61 (1.05–2.46)**
Aortic involvement		1.39 (0.85–2.27)		**1.97 (1.19–3.27)**
Coronary artery disease		**2.83 (1.84–4.42)**		**2.30 (1.48–3.59)**
Heart failure		**3.19 (2.21–4.60)**		**2.40 (1.64–3.54)**
Cerebrovascular disease		1.52 (0.97–2.38)		1.31 (0.83–2.05)
Cardiovascular disease, any		**2.82 (1.59–4.07)**		**2.22 (1.52–3.24)**
Diabetes mellitus, type I		**1.92 (1.01–3.67)**		**1.97 (1.03–3.78)**
Diabetes type II		1.44 (0.92–2.25)		**1.69 (1.08–2.66)**
Diabetes mellitus, any		**2.03 (1.31–3.15)**		**1.81 (1.17–2.82)**
Chronic pulmonary disease		**2.38 (1.50–3.79)**		**2.19 (1.38–3.48)**
Chronic kidney disease with renal failure		**4.40 (2.30–8.43)**		**3.39 (1.75–6.58)**
Renal failure unspecified		**3.21 (1.68–6.13)**		**2.51 (1.31–4.82)**
Malignancy (solid tumors)		1.24 (0.65–2.37)		1.20 (0.63–2.30)
Hip fracture		1.36 (0.79–2.34)	0.80 (0.46–1.38)	
Vertebral fracture		1.54 (0.57–4.19)	0.97 (0.36–2.62)	
Wrist fracture		0.77 (0.37–1.57)	0.63 (0.31–1.29)	
Proximal humeral fracture		2.03 (0.99–4.16)	**2.16 (1.05–4.44)**	

GCA: Giant cell arteritis; HR: Hazard ratio; CI: Confidence interval; SD: Standard deviation; NA: Not applicable

Bold text indicates statistically significant findings

The LVI phenotype was not a significant predictor in the crude (unadjusted) analysis. However, in age-adjusted models, both LVI overall (hazard ratio (HR) 1.61; 95% CI 1.05; 2.46) and aortic involvement with or without distributary involvement (HR 1.97; 95% CI 1.19; 3.27) reached statistical significance as predictors for future severe infection. Exploratory analyses revealed significantly increased risk (unadjusted and age-adjusted) also in those with visceral artery involvement, but there was no association with LVI of cranial arteries or extremity arteries (Supplementary Table 7).

In further exploratory analyses including subtypes of LVI, there was an association for ectasia with severe infections, and a similar trend for aneurysm in age-adjusted analyses, but no significant associations for stenosis, dissection or positive PET-CT/MRI indicating vascular inflammation (Supplementary Table 8).

Higher CCI was not significantly associated with severe infections (Table 4). A high CCI was mainly driven by prior diagnoses of solid tumor malignancies, which were not predictive of severe infections (Table 4).

As shown in Table 4, several prevalent comorbidities at GCA diagnosis were associated with subsequent severe infections in crude and age-adjusted analyses. In the final multivariable model, aortic involvement was significantly predictive of severe infections, adjusted for age, sex, diabetes, chronic obstructive pulmonary disease, chronic kidney disease with renal failure and proximal humeral fracture (Table 5).

**Table 5:** Multivariable analysis of potential risk factors for severe infections in patients with GCA

Cox regression; multivariable analysis*	
Variables	HR (95% CI)
Age, per 5 years	**1.44 (1.25–1.64)**
Male sex	1.45 (0.97–2.18)
Aortic involvement	**1.75 (1.04–2.94)**
Diabetes	**1.82 (1.16–2.84)**
Chronic obstructive pulmonary disease	**1.89 (1.18–3.03)**
Chronic kidney disease with renal failure	**2.90 (1.48–5.67)**
Proximal humeral fracture	**2.29 (1.08–4.86)**

GCA: Giant cell arteritis

* Includes all variables listed in the table

Bold text indicates statistically significant findings

Results were largely similar in three alternative models, where chronic kidney disease with renal failure was replaced by cardiovascular disease (all combined), coronary artery disease and heart failure, respectively (Supplementary Table 9).

## Discussion

In this study, we found higher rates of severe infections during the first six months after GCA diagnosis compared to the general population. LVI, in particular aortic involvement, was predictive of severe infections. Higher age and several comorbidities were also associated with increased infection risk, but the association with involvement of the aorta remained significant in multivariate analysis, suggesting that this phenotype has an independent impact on the risk of severe infections. Arterial ectasia and possibly also aneurysms, but not stenosis, may have a particular impact on infection risk, and there may also be an association with LVI of the visceral arteries.

The increased risk of severe infections in patients with GCA compared to the general populations is in agreement with most [19–22, 30], but not all [21], previous studies. Several other studies have also reported the highest rate of infections early during the disease course [20–22]. Our rate ratio for the first 6 months after diagnosis of 3.27 is in agreement with the findings by Durand et al. (3.57 for months 0–5) [22] and by Schmidt et al. (2.1 for the first year) [20]. We found that the risk remained moderately elevated after more than two years of follow-up. The increased risk of severe infections may reflect immunosuppressive effects of treatment or of the disease itself. Interestingly it has been shown that patients with GCA have an increased rate of infections prior to diagnosis [31, 32], possibly suggesting a constitutionally increased susceptibility to infections in individuals developing GCA, although it is difficult to exclude underlying misclassification of infections or protopathic bias [32]. We observed a similar trend for severe infections prior to diagnosis in the present study.

The observation that pre-existing comorbidities, i.e. diabetes, chronic obstructive pulmonary disease, chronic kidney disease with renal failure, CVD, heart failure and proximal humeral fracture, were predictive of severe infections in patients with GCA, independently of age, is also compatible with the literature. For example, a recent study of deaths caused by infection in the general population demonstrated associations with most of these comorbidities [33]. Regarding proximal humeral fractures, a recent nationwide study from Denmark showed that this was one of the major osteoporotic fracture types with a strong association with impaired survival, and that a major proportion of the excess mortality was mediated by pneumonia and other severe infections [34]. The present results suggest that proximal humeral fracture may be particularly related to frailty in patients with a chronic disease such as GCA. As we studied comorbidities that were present before diagnosis, such fractures could reflect pre-existing osteoporosis in a subset of patients that develop GCA.

Disease phenotype as predictor for severe infections has, to our best knowledge, not been investigated hitherto. Some previous studies have shown the presence of LVI in GCA to be associated with higher relapse frequency [23–25], which could affect infection risk. In general, inflammation increases the baseline level of infection susceptibility [35–37]. It stands to reason that GCA patients with higher level of inflammatory burden, e.g. those with widespread vasculitis or vasculitis of the largest arteries, i.e. aorta and/or its main branches, are more prone to infection. The high RR during the first six months is compatible with an early effect of active inflammation and/or high GC doses over time in early GCA on the risk of severe infections. The HR of LVI as risk factor for severe infection increased after adjusting for age. This could be explained by the inverse association between age and LVI in patients with GCA [27, 38, 39]. Taking the younger age of LVI patients into account thus enables a more accurate estimate of the impact of the LVI phenotype on infection risk.

The lack of association between GC dose at diagnosis and subsequent severe infections may be due to the practice of using high-dose GC in the vast majority of patients. The substantial amount of missing data considering this variable may have impacted the analysis.

The most common types of severe infections caused by bacteria in patients with GCA were pneumonia, urinary tract infections and septicemia. This pattern of infections that are frequent in the community is in agreement with previous studies of patients with GCA [19, 22]. We did not identify any major contribution from opportunistic infections, in particular there were no infections with Pneumocystis jirovecii. Prophylactic treatment for pneumocystis pneumonia in patients with GC monotherapy for GCA is not standard practice in Sweden. Taken together, this suggests that such infections do not constitute a major problem in patients with GCA.

We investigated inpatient episodes, which are more likely to be clinically relevant, but we cannot exclude that patients with GCA might be more likely to be hospitalized due to ongoing immunosuppressive treatment.

Limitations are related to the retrospective study design. Data on GC doses over time after diagnosis and other immunosuppressive treatment (e.g. methotrexate), and data on relapses, were not available. In particular, cumulative GC dose is a key determinant of infections, and would have been of interest. Further prospective studies should investigate the impact of these factors on infection risk. Patients were followed according to standard of care, with no structured follow-up protocol. As no systematic standardized vascular imaging studies were performed at diagnosis or later on, due to the clinical practice at the time of the study, the proportion with LVI is likely underestimated. Recent prospective imaging studies have reported LVI in 29% − 83% of GCA patients [3, 40, 41]. Many patients did not undergo any relevant imaging study, and the number of patients with imaging findings indicating current inflammation was too small for meaningful analyses. Due to the lack of relevant imaging in almost 2/3 of the patients, the results should be taken with caution. Furthermore, with the present definition, LVI includes all types of vessel involvement, and does not require specific demonstration of vasculitis. Therefore, we cannot exclude that atherosclerosis may have contributed to some LVI lesions. Data on LVI in the reference subjects, and on CVD risk factors beyond age, sex and diabetes, were not available. Another limitation is lack of data regarding vaccination. This information was difficult to obtain retrospectively in the available records and was thus not collected. Due to the establishment of the SHR in 1998, the maximum period for retrieval of prior comorbidities for those diagnosed in 2002 was 4 years. As the definitions of comorbidities and of the outcome severe infection were based on registered diagnoses only, with no individual validation, we cannot exclude that misclassification may occur. However, we do not expect a differential effect of misclassification in patients with GCA and reference subjects. Furthermore, the results are only applicable to patients with biopsy-positive GCA. We cannot exclude that LVI and other factors could have a different impact on infection risk in biopsy-negative GCA. Finally, available follow-up data were limited and did not include the most recent decade. Management of GCA has changed to some extent, although monotherapy with GC has continued to be the first treatment choice in most patients. Therefore, the data on early infection risk are likely relevant to current practice. However, it would be of interest to investigate these aspects in a more recent time period.

Strengths include the population-based investigation of a regional cohort in a country with a high incidence of GCA. As our study only included patients with biopsy-proven GCA, diagnoses should be valid. The sample size was relatively large, with an extensive follow-up time. There was detailed information on baseline comorbidities, obtained in a consistent manner for all patients with GCA.

## Conclusions

In conclusion, patients with biopsy-positive GCA have increased risk of severe infections, in particular in early disease, close to diagnosis. In addition to age and comorbidities, LVI, especially aortic involvement, may predict severe infections in GCA, although further prospective studies including detailed assessment of LVI are necessary. Based on these results, particular vigilance regarding infections and adequate preventive strategies, including vaccination, in this subset of patients with GCA is recommended.

## Supplementary Information

Below is the link to the electronic supplementary material.

Supplementary Material 1: Supplementary Figure 1 Incidence rate ratios (IRR), with 95% confidence intervals, for first severe infections in each category of follow-up, in patients with GCA vs reference subjects.

Supplementary Material 2

## Data Availability

The datasets used and/or analyzed during the current study are available from the corresponding author on reasonable request.

## Abbreviations

CCI: Charlson comorbidity index
CI: Confidence interval
CVD: Cardiovascular disease
GC: Glucocorticoids
GCA: Giant cell arteritis
HR: Hazard ratio
ICD-10: The international classification of diseases, tenth revision, clinical modification
IR: Incidence rate
IRR: Incidence rate ratio
LVI: Large vessel involvement
18-FDG PET-CT: 18-fluoro-2-deoxy-d-glucose positron emission tomography–computed tomography
PY: Person-years
RR: Rate ratio
SD: Standard deviation
SHR: Skåne healthcare register
SPSS: Statistical package for the social sciences
