# Supplementary figures and images for: Severe infections in giant cell arteritis - incidence over time and relation to large vessel involvement and comorbidities, a population-based study
Source: BMC Rheumatol. 2026 Jul 22;10:60. doi: 10.1186/s41927-026-00676-2 (PMC13393624; doi:10.1186/s41927-026-00676-2)

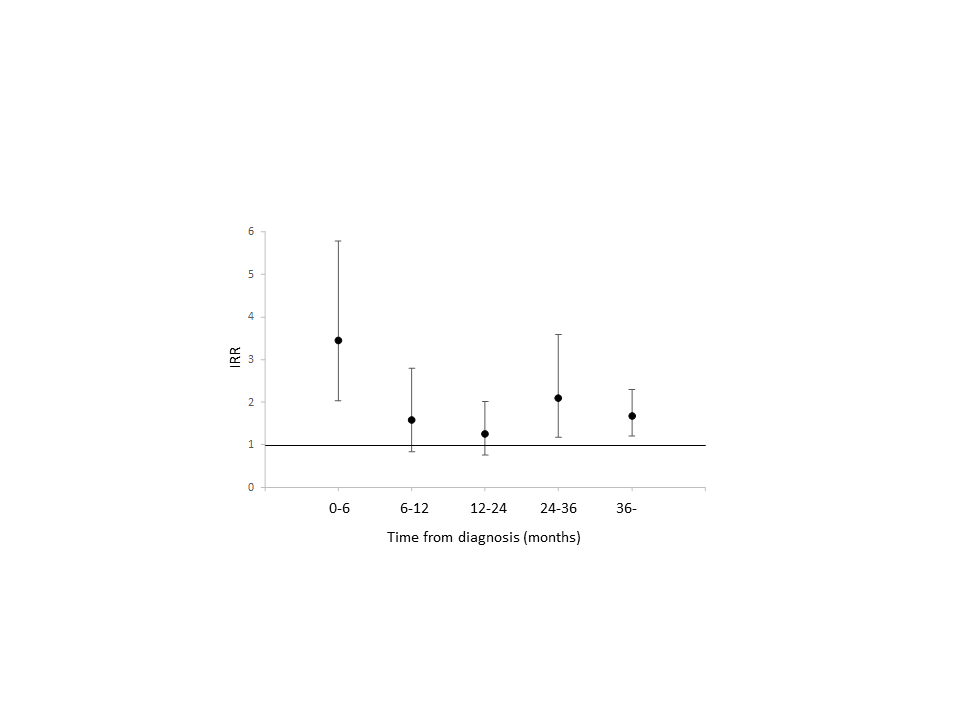

Supplement: Supplementary file 1 — Supplementary Material 1: Supplementary Figure 1 Incidence rate ratios (IRR), with 95% confidence intervals, for first severe infections in each category of follow-up, in patients with GCA vs reference subjects. [file 41927_2026_676_MOESM1_ESM.tif]
