# Supplementary material for: Severe infections in giant cell arteritis - incidence over time and relation to large vessel involvement and comorbidities, a population-based study
Source: BMC Rheumatol. 2026 Jul 22;10:60. doi: 10.1186/s41927-026-00676-2 (PMC13393624; doi:10.1186/s41927-026-00676-2)
Supplement: Supplementary file 2 — Supplementary Material 2 [file 41927_2026_676_MOESM2_ESM.docx]

# Supplementary material

**Supplementary Table 1.** Comorbidities defined by ICD-10 codes

| **Comorbidity** | **ICD-10 codes** |
| --- | --- |
| **Cardiovascular disease** |  |
| Coronary artery disease | I21, I22, I23, I24, I25 |
| Heart failure | I50 |
| Cerebrovascular disease | I61, I62, I63, I64, I65, I66, |
| Peripheral arterial disease | I70, I71, I72,  I739B, I739C, I739X |
| **Diabetes mellitus** |  |
| Type I | E10 |
| Type II | E11 |
| **Pulmonary disease** |  |
| Chronic obstructive pulmonary disease | J44 |
| **Kidney disease** |  |
| Chronic kidney disease with renal failure | N18 |
| Renal failure, non-specified | N19 |
| **Osteoporosis related fractures** |  |
| Hip fracture | S72.0, S72.1, S72.2 |
| Vertebral fracture | S22.0, S22.1, S32.0, S32.7 |
| Wrist fracture | S52.0, S52.5, S52.8 |
| Proximal humerus fracture | S42.2 |

**Supplementary Table 2**. List of investigated infection types, defined by ICD-10 codes

| **ICD-10 codes, infections** | | |
| --- | --- | --- |
| **Nr** | **ICD-10 codes** | **Infection types** |
| 1 | A09 | Gastroenteritis |
| 2 | B34 | Viral infections |
| 3 | A40-A41 | Septicaemia |
| 4 | B99 | Non-specific infection |
| 5 | A08 | Viral gastroenteritis |
| 6 | A04 | Other bacterial gastroenteritis |
| 7 | A87 | Viral meningitis |
| 8 | A49 | Bacterial infection with un-specified localisation |
| 9 | B27 | Mononucleosis |
| 10 | B02 | Zoster |
| 11 | B15 | Acute hepatitis A |
| 12 | B19 | Non-specified viral hepatitis with coma |
| 13 | B95 | Streptococci and staphylococci as cause of disease otherwise classified |
| 14 | B97 | Virus as cause of infection otherwise classified |
| 15 | L00-L08 | Skin infections |
| 16 | J00-J06 | Acute infections in **u**pper **r**espiratory **t**racts (URT) |
| 17 | J09-J18 | Influenza and pneumonias |
| 18 | J20-J22 | Other acute infections in **l**ower **r**espiratory **t**racts (LRT) |
| 19 | J85-J86 | Pyogenic infections in lower respiratory tracts |
| 20 | M00-M01 | Septic arthritis/Infectious joint diseases |

**Supplementary Table 3.** The distribution of affected vessels in patients
with large vessel involvement.

| **Vascular bed** | **Number (%)** |
| --- | --- |
| Aorta ascendens |  |
| Aneurysm | 21 (4.1) |
| Ectasia | 7 (1.4) |
| Vasculitis | 5 (1.0) |
| Aorta descendens |  |
| Aneurysm | 3 (0.6) |
| Ectasia | 10 (1.9) |
| Vasculitis | 2 (0.4) |
| Abdominal aorta |  |
| Aneurysm | 20 (3.9) |
| Ectasia | 10 (1.9) |
| Vasculitis | 2 (0.4) |
| Aortic dissections | 6 (1.2) |
| Intracranial arteries | 7 (1.4) |
| Carotid arteries |  |
| Stenosis/occlusion | 18 (3.5) |
| Ectasia/aneurysm | 2 (0.4) |
| Vasculitis | 4 (0.8) |
| Vertebral arteries |  |
| Stenosis/occlusion | 2 (0.4) |
| Vasculitis | 2 (0.4) |
| Brachiocephalic trunc |  |
| Ectasia/aneurysm | 1 (0.2) |
| Vasculitis | 4 (0.8) |
| Subclavian arteries |  |
| Stenosis/occlusion | 1 (0.2) |
| Ectasia/aneurysm | 1 (0.2) |
| Vasculitis | 4 (0.8) |
| Axillary arteries |  |
| Stenosis/occlusion | 1 (0.2) |
| Vasculitis | 1 (0.2) |
| Pulmonary arteries |  |
| Ectasia/aneurysm | 1 (0.2) |
| Coeliac trunc |  |
| Stenosis/occlusion | 2 (0.4) |
| Superior mesenteric artery |  |
| Stenosis/occlusion | 3 (0.6) |
| Inferior mesenteric artery |  |
| Stenosis/occlusion | 1 (0.2) |
| Renal arteries |  |
| Stenosis/occlusion | 5 (1.0) |
| Iliac arteries |  |
| Stenosis/occlusion | 5 (1.0) |
| Ectasia/aneurysm | 4 (0.8) |
| Femoral arteries |  |
| Stenosis/occlusion | 12 (2.3) |
| Vasculitis | 2 (0.4) |
| Lower leg arteries |  |
| Stenosis/occlusion | 10 (1.9) |

**Supplementary Table 4.** Rates of first severe infection in patients with giant cell arteritis and reference subjects

|  | **Cases** | | | **Reference subjects** | | |
| --- | --- | --- | --- | --- | --- | --- |
| Follow-up interval (months) | Subjects with first infection (n) | Follow-up  (py) | Rate/100 py  (95% CI) | Subjects with first infection (n) | Follow-up  (py) | Rate/100 py (95% CI) |
| 0-6 | 29 | 244.52 | 11.9  (7.9; 17.0) | 34 | 990.41 | 3.3  (2.4; 4.8) |
| 6-12 | 17 | 236.03 | 7.2  (4.2; 11.5) | 44 | 962.93 | 4.6  (3.3; 6.1) |
| 12-24 | 23 | 431.95 | 5.32 (3.4; 8.0) | 75 | 1777.20 | 4.2  (3.3; 5.3) |
| 24-36 | 21 | 349.20 | 6.0  (3.7; 9.2) | 42 | 1465.19 | 2.9  (2.1; 3.9) |
| 36- | 56 | 933.16 | 6.0  (4.5; 7.8) | 142 | 3963.04 | 3.6  (3.0; 4.2) |

n: number; py: person-years; CI: confidence interval; * First during each time interval.

**Supplementary Table 5.** Pathogens in infections verified by
culture §

| **Gram negative rods** | N= 39 |
| --- | --- |
| Escherichia Coli | 21 |
| Pseudomonas Aeruginosa | 5 |
| Others | 13* |
| **Gram positive cocci** | N=22 |
| Staphylococci | 11 |
| Staphylococcus Aureus | 10 |
| Staphylococcus species | 1 |
| Streptococci | 4 |
| Streptococcus Pneumoniae | 2 |
| α- streptococcus | 1 |
| Group A β- streptococcus | 1 |
| Enterococcus Faecalis | 7 |
| **Clostridium Difficile** | N=9 |
| **Other bacteria** | N=2# |

********:* Bacteroides Fragilis n=2, Bacteroides Freundii n=2, Proteus species n=1, Proteus Mirabilis n=2, Serratia Marcescens n=1, Klebsiella Oxytoca n=2, Klebsiella Pneumonie n=3.
***#****:* Moraxella Catarrhalis, Mycoplasma Pneumonie.
**§**: n=11 patients had several pathogens in their cultures (one each per combination):
S. Aureus + Enterococcus Faecalis + group A β- streptococcus,
S. Aureus + Enterococcus Faecalis + Bacteroides Fragilis,
S. Aureus + group A β- streptococcus,
S. Aureus + Gram negative mixed flora,
Bacteroides Fragilis + Citrobacter Freundii,
Enterococcus Faecalis + Klebsiella Pneumonie,
Enterococcus Faecalis + Enterococcus Cloacae + Streptococcus Pneumonie,
Escherichia Coli + Clostridium Difficile,
Pseudomonas + Candida Glabrata,
α- streptococcus + Candida Albicans,
Skin lesion with rich faecal flora with enterococci + gram negative rods + urine culture with gram positive mixed flora.

**Supplementary Table 6.** Incidence of severe infections before and after diagnosis date/index date in GCA cases and controls

|  | **Cases** | | | **Controls** | | |
| --- | --- | --- | --- | --- | --- | --- |
|  | Infections (n) | Follow-up  (py) | Rate/100 py  (95% CI) | Infections (n) | Follow-up  (py) | Rate/100 py  (95% CI) |
| Before diagnosis /index date | 103 | 4413 | 2.3  (1.9; 2.8) | 331 | 17509 | 1.9  (1.7; 2.1) |
| After diagnosis / index date | 221 | 2300 | 9.6  (8.4; 11.0) | 576 | 9418 | 6.1  (5.6; 6.6) |

**Supplementary Table 7**. Relation between arterial involvement and severe infections in patients with giant cell arteritis. Cox regression

| **Arterial involvement** | **n (%)** | **Crude**  **HR (95% CI)** | **Age-adjusted**  **HR (95% CI)** |
| --- | --- | --- | --- |
| Cranial arteries^1^ | 32 (6.2) | 0.89 (0.41-1.91) | 1.04 (0.48-2.25) |
| Upper extremity arteries^2^ | 7 (1.4) | 0.49 (0.07-3.52) | 0.80 (0.11-5.85) |
| Aorta | 46 (8.9) | 1.39 (0.85 - 2.27) | **1.97 (1.19 - 3.27)** |
| Visceral arteries^3^ | 9 (1.7) | **3.68 (1.49-9.03)** | **4.77 (1.92-11.8)** |
| Lower extremity arteries^4^ | 22 (4.3) | 0.99 (0.40-2.43) | 0.89 (0.36-2.19) |

HR: Hazard ratio; CI: Confidence interval

^1^ Intracranial, carotid, vertebral or brachiocephalic arteries

^2^ Subclavian or axillary arteries

^3^ Pulmonary, mesenteric, celiac or renal arteries

^4^ Iliac, femoral, popliteal or tibial arteries

**Supplementary Table 8**. Relation between type of large vessel involvement and severe infections in patients with giant cell arteritis. Cox regression

| **Subtype** | **n (%)** | **Crude**  **HR (95% CI)** | **Age-adjusted**  **HR (95% CI)** |
| --- | --- | --- | --- |
| Aneurysm | 39 (7.6) | 1.06 (0.55-2.02) | 1.59 (0.82-3.09) |
| Ectasia | 25 (4.8) | **2.11 (1.10-4.05)** | **2.41 (1.25-4.62)** |
| Stenosis | 41 (7.9) | 1.04 (0.52-1.92) | 1.05 (0.55-2.00) |
| Dissection | 6 (1.2) | 0.60 (0.08-4.32) | 1.00 (0.14-7.23) |
| Positive PET-CT/MRI | 6 (1.2) | 1.58 (0.39-6.38) | 2.80 (0.68-11.6) |

**Supplementary Table 9.** Alternative multivariable models of potential risk
factors for severe infections in patients with GCA. Cox regression

| **Variables** | **Model 1***  **HR (95% CI)** | **Model 2***  **HR (95% CI)** | **Model 3***  **HR (95% CI)** |
| --- | --- | --- | --- |
| Age, per 5 years | **1.39 (1.21-1.59)** | **1.44 (1.26-1.65)** | **1.38 (1.21-1.58)** |
| Male sex | 1.47 (0.98-2.20) | 1.33 (0.88-2.02) | 1.49 (1.00-2.24) |
| Aortic involvement | 1.68 (0.99-2.84) | **1.76 (1.04-2.98)** | 1.65 (0.98-2.78) |
| Diabetes | **1.70 (1.09-2.67)** | **1.82 (1.17-2.85)** | **1.66 (1.05-2.60)** |
| Chronic obstructive pulmonary disease | **1.66 (1.03-2.68)** | **1.79 (1.11-2.89)** | 1.57 (0.97-2.59) |
| Proximal humeral fracture | **2.45 (1.16-5.16)** | **2.14 (1.01-4.52)** | **2.36 (1.12-4.98)** |
| Cardiovascular disease | **1.95 (1.32-2.56)** | NI | NI |
| Coronary artery disease | NI | **1.98 (1.25-3.13)** | NI |
| Heart failure | NI | NI | **2.02 (1.36-3.00)** |

GCA: Giant cell arteritis; NI: Not included

* Includes all variables listed in the column.

Bold text indicates statistically significant findings
